# Supplementary material for: Psychometric and biomedical outcomes of glycated haemoglobin target-setting in adults with type 1 and type 2 diabetes: Protocol for a mixed-methods parallel-group randomised feasibility study
Source: PLoS One. 2022 Oct 27;17(10):e0275980. doi: 10.1371/journal.pone.0275980 (PMC9612465; doi:10.1371/journal.pone.0275980)
Supplement: S1 File — (DOCX) [file pone.0275980.s002.docx]

**Biological specimen usage**

Extracted from HRA IRAS application form. IRAS Project ID: **291254**

1. **What types of human tissue or other biological material will be included in the study?**

Capillary blood sampling via skin puncture with a lancet (“finger-pricking”) for point-of-care testing of glycated haemoglobin (HbA1c). Samples will be disposed of once analysed by the point-of-care HbA1c machine.

1. **Who will collect the samples?**

The St Helens and Knowsley Teaching Hospitals NHS Trust (STHK) diabetes centre clinic nurse or healthcare assistant.

1. **Who will the samples be removed from?**

Living donors

1. **Will informed consent be obtained from living donors for use of the samples?**

**In this research?**

Yes

**In future research?**

Yes

1. **Will any tissues or cells be used for human application or to carry out testing for human application in this research?**

No

1. **Will the samples be stored?**

**In fully anonymised form?**

No

**In linked anonymised form? (linked to stored tissue but donor not identifiable to researchers)**

No

**In a form in which the donor could be identifiable to researchers?**

No

1. **What types of test or analysis will be carried out on the samples?**

A point-of-care analysis of capillary blood to obtain a glycated haemoglobin (HbA1c) reading.

This test is performed routinely to aid management decisions in people with diabetes. This test will be carried out as part of normal diabetes clinic processes in order to aid the continued optimisation of participant's management by diabetes clinicians external to the research. Analysis of the data will also review the potential impact that the study intervention has on participants' HbA1c readings. The HbA1c readings will be used as part of the intervention delivered in the study: the evaluation of the preliminary impact that setting HbA1c goals (against participants' pre-existing HbA1c readings) has on their well-being (measured using a series of validated psychometric questionnaires).

1. **Will the research involve the analysis or use of human DNA in the samples?**

No

1. **Is it possible that the research could produce findings of clinical significance for donors or their relatives?**

Yes

1. **If so, will arrangements be made to notify the individuals concerned?**

Yes

**If No, please justify. If Yes, say what arrangements will be made and give details of the support or counselling service.**

Individuals will be notified of their HbA1c readings throughout the study.

Use of capillary blood HbA1c readings is an important process in the study intervention: Setting explicit HbA1c targets above or below participants' current HbA1c readings. A leaflet will be provided to participants explaining what HbA1c is in the context of their diabetes. This leaflet will display their current HbA1c reading against a target HbA1c set for them. Participants will have the opportunity to discuss their HbA1c with the researcher. The participant will also attend diabetes clinic appointments with a senior diabetes clinician at baseline and endpoint of the feasibility study.

1. **Give details of where the samples will be stored, who will have access and the custodial arrangements.**

Samples will not be stored. Upon completion of point-of-care sample analysis, samples will be safely disposed of on-site in the diabetes centre.

1. **What will happen to the samples at the end of the research? Please tick all that apply and give further details.**

Disposal in accordance with the Human Tissue Authority’s Code of Practice.
