# Supplementary material for: Psychometric and biomedical outcomes of glycated haemoglobin target-setting in adults with type 1 and type 2 diabetes: Protocol for a mixed-methods parallel-group randomised feasibility study
Source: PLoS One. 2022 Oct 27;17(10):e0275980. doi: 10.1371/journal.pone.0275980 (PMC9612465; doi:10.1371/journal.pone.0275980)
Supplement: Attachment — Submitted filename: Response to reviewers.docx [file pone.0275980.s004.docx]

14^th^ May 2022

**Re: PLOS ONE Decision: Revision required [PONE-D-21-33122] - [EMID:c1e2bb034c361eaf]**

Dear Editor and Reviewers,

On behalf of my co-authors, I would like to thank you for reviewing our article entitled “Psychometric and biomedical outcomes of glycated haemoglobin target-setting in adults with type 1 and type 2 diabetes: Protocol for a mixed-methods parallel-group randomised feasibility study” with opportunity to revise and resubmit.

We found the reviewer’s comments helpful in revising the manuscript and have carefully considered their suggestions. We revised the manuscript in accordance with the reviewer’s comments and the journal requirements.

Below, we have outlined journal requirements and reviewer comments in black followed by our responses in red, prefaced “Author response”. Corresponding changes are highlighted in the tracked manuscript text file in red using the ‘track changes’ function.

Yours Sincerely,

Journal requirements **and** author responses

1. Please ensure that your manuscript meets PLOS ONE's style requirements, including those for file naming.

Author response:

We have reformatted the manuscript to comply with PLOS ONE’s style requirements. Changes are marked using the ‘tracked changes’ function. Additionally, the table presented in Fig 1 has been formatted to comply with the journal style requirements and inserted in-line in the manuscript as ‘Table 1’ in the appropriate place. Other table and figure numerical assignments have been updated accordingly.

2. We note that the grant information you provided in the ‘Funding Information’ and ‘Financial Disclosure’ sections do not match.

Author response:

We have amended the funding information section (see point 3).

3. Thank you for stating the following in the Acknowledgments Section of your manuscript: "The authors thank Mrs Jeanette Anders for assistance with ethical approval and sponsorship of the research, Dr Niall Furlong for advice and support and Dr Sumudu Bujawansa for pastoral support."

We note that you have provided funding information that is not currently declared in your Funding Statement. However, funding information should not appear in the Acknowledgments section or other areas of your manuscript. We will only publish funding information present in the Funding Statement section of the online submission form. Please remove any funding-related text from the manuscript and let us know how you would like to update your Funding Statement. Currently, your Funding Statement reads as follows: "The corresponding author (S.J.W.) is a PhD student funded by the Department of Diabetes and Endocrinology, St Helens and Knowsley Teaching Hospitals NHS Trust (STHK). The study is sponsored by STHK (sponsorship reference STHK-2021-003). STHK are not involved in the study design, the preparation of this paper or the decision to submit the paper for publication. The funding body will not be involved in data collection, analysis or interpretation. St Helens and Knowsley Teaching Hospitals NHS Trust (STHK): https://www.sthk.nhs.uk"

Author response:

Apologies for the confusion. The authors received no specific grants to undertake this research. We would like to update the following sections:

Acknowledgements update: “This work forms part of the first authors PhD. S.J.W. is supported by the Department of Diabetes and Endocrinology, St Helens and Knowsley Teaching Hospitals NHS Trust, UK.” (updated in manuscript using the ‘tracked changes’ function)

Financial disclosure/funding information: “The authors received no specific funding for this work.”

4. In your Data Availability statement, you have not specified where the minimal data set underlying the results described in your manuscript can be found. PLOS defines a study's minimal data set as the underlying data used to reach the conclusions drawn in the manuscript and any additional data required to replicate the reported study findings in their entirety. All PLOS journals require that the minimal data set be made fully available. For more information about our data policy, please see <http://journals.plos.org/plosone/s/data-availability>.

Author response:

We would like to update our data availability statement with the following text:

“All relevant data from this study will be made available on study completion. Anonymised data will be deposited in the Edge Hill University online research data repository, <https://figshare.edgehill.ac.uk>.”

5. Please note that in order to use the direct billing option the corresponding author must be affiliated with the chosen institute. Please either amend your manuscript to change the affiliation or corresponding author, or email us at plosone@plos.org with a request to remove this option.

Author response:

The corresponding author is affiliated with both Edge Hill University (who are a member of the PLOS Institutional Account Program) and St Helens and Knowsley Teaching Hospitals NHS Trust. The corresponding author has altered the order in which these affiliations are listed to put Edge Hill University first.

6. Please review your reference list to ensure that it is complete and correct. If you have cited papers that have been retracted, please include the rationale for doing so in the manuscript text, or remove these references and replace them with relevant current references. Any changes to the reference list should be mentioned in the rebuttal letter that accompanies your revised manuscript. If you need to cite a retracted article, indicate the article’s retracted status in the References list and also include a citation and full reference for the retraction notice.

Author response:

Reference list reviewed:

1. Reference 14 added as an update (a correction to a prior paper). The correction article notes it should be considered in conjunction with the original article.
2. Reference 15 url added.
3. Reference 21 url added.
4. Reference 28 url added.
5. Reference 30 url added.
6. Reference 52 doi added.
7. Reference 53 doi added.
8. Reference 57 doi added.

Reviewers' comments **and** author responses:

1. Does the manuscript provide a valid rationale for the proposed study, with clearly identified and justified research questions?

Reviewer #1: Yes

2. Is the protocol technically sound and planned in a manner that will lead to a meaningful outcome and allow testing the stated hypotheses?

Reviewer #1: Yes

3. Is the methodology feasible and described in sufficient detail to allow the work to be replicable?

Reviewer #1: Yes

4. Have the authors described where all data underlying the findings will be made available when the study is complete?

Reviewer #1: Yes

5. Is the manuscript presented in an intelligible fashion and written in standard English?

Reviewer #1: Yes

6. Review Comments to the Author

Reviewer #1: This protocol paper was a pleasure to read and is exploring a very important yet under researched topic of how clinician-initiated HbA1c testing and setting A1c targets affects the wellbeing and self-care of people living with diabetes. The manuscript was easy to read and the introduction had a strong rationale leading to a good description of the methods and analyses. The mixed-methods design is appropriate. My only concern was the statistical analysis section. As this is a feasibility study and underpowered the authors should not be conducting any statistical tests (e.g. t-tests) looking for differences from baseline to post-intervention but rather simply reporting means and SDs. Also the qualitative analysis section was very brief.

Author response:

Thank you. We agree with the reviewer’s comments regarding statistical analysis and have adjusted the manuscript accordingly. We have also expanded the qualitative analysis section with additional detail. (Pages 15 and 16, lines 328–354)

7. PLOS authors have the option to publish the peer review history of their article (what does this mean?). If published, this will include your full peer review and any attached files.

If you choose “no”, your identity will remain anonymous but your review may still be made public.

Do you want your identity to be public for this peer review? For information about this choice, including consent withdrawal, please see our Privacy Policy.

Reviewer #1: No

------END OF RESPONSE------
