# Supplementary material for: Psychometric and biomedical outcomes of glycated haemoglobin target-setting in adults with type 1 and type 2 diabetes: Protocol for a mixed-methods parallel-group randomised feasibility study
Source: PLoS One. 2022 Oct 27;17(10):e0275980. doi: 10.1371/journal.pone.0275980 (PMC9612465; doi:10.1371/journal.pone.0275980)
Supplement: Attachment — Submitted filename: Response to reviewers.docx [file pone.0275980.s005.docx]

14^th^ August 2022

**Re: PLOS ONE Decision: Revision required [PONE-D-21-33122] - [EMID:c1e2bb034c361eaf]**

Dear Editor and Reviewers,

On behalf of my co-authors, I would like to thank you for reviewing our article entitled “Psychometric and biomedical outcomes of glycated haemoglobin target-setting in adults with type 1 and type 2 diabetes: Protocol for a mixed-methods parallel-group randomised feasibility study” with opportunity to revise and resubmit.

We found the reviewer’s comments helpful in revising the manuscript and have carefully considered their suggestions. We revised the manuscript in accordance with the reviewer’s comments.

Below, we have outlined reviewer comments in black followed by our responses in red, prefaced “Author response”. Corresponding changes are highlighted in the tracked manuscript text file in red using the ‘track changes’ function.

Yours Sincerely,

Review comments **and** author responses

Reviewers' comments:

Reviewer's Responses to Questions

**Comments to the Author**

1. Does the manuscript provide a valid rationale for the proposed study, with clearly identified and justified research questions?

The research question outlined is expected to address a valid academic problem or topic and contribute to the base of knowledge in the field.

Reviewer #2: Yes

2. Is the protocol technically sound and planned in a manner that will lead to a meaningful outcome and allow testing the stated hypotheses?

The manuscript should describe the methods in sufficient detail to prevent undisclosed flexibility in the experimental procedure or analysis pipeline, including sufficient outcome-neutral conditions (e.g. necessary controls, absence of floor or ceiling effects) to test the proposed hypotheses and a statistical power analysis where applicable. As there may be aspects of the methodology and analysis which can only be refined once the work is undertaken, authors should outline potential assumptions and explicitly describe what aspects of the proposed analyses, if any, are exploratory.

Reviewer #2: Yes

3. Is the methodology feasible and described in sufficient detail to allow the work to be replicable?


Descriptions of methods and materials in the protocol should be reported in sufficient detail for another researcher to reproduce all experiments and analyses. The protocol should describe the appropriate controls, sample size calculations, and replication needed to ensure that the data are robust and reproducible.

Reviewer #2: Yes

4. Have the authors described where all data underlying the findings will be made available when the study is complete?

The [PLOS Data policy](https://eur01.safelinks.protection.outlook.com/?url=https%3A%2F%2Fjournals.plos.org%2Fplosone%2Fs%2Fmaterials-and-software-sharing&data=05%7C01%7C23723122%40edgehill.ac.uk%7Cdbeec7f19a7d4497e44608da6e659082%7C093586914d8e491caa760a5cbd5ba734%7C0%7C0%7C637943679743277065%7CUnknown%7CTWFpbGZsb3d8eyJWIjoiMC4wLjAwMDAiLCJQIjoiV2luMzIiLCJBTiI6Ik1haWwiLCJXVCI6Mn0%3D%7C3000%7C%7C%7C&sdata=NhcHlO934V4%2FTZmosZG4C%2FpUUoMz666c0XPQROrQMn4%3D&reserved=0) requires authors to make all data underlying the findings described in their manuscript fully available without restriction, with rare exception, at the time of publication. The data should be provided as part of the manuscript or its supporting information, or deposited to a public repository. For example, in addition to summary statistics, the data points behind means, medians and variance measures should be available. If there are restrictions on publicly sharing data—e.g. participant privacy or use of data from a third party—those must be specified.

Reviewer #2: Yes

5. Is the manuscript presented in an intelligible fashion and written in standard English?

PLOS ONE does not copyedit accepted manuscripts, so the language in submitted articles must be clear, correct, and unambiguous. Any typographical or grammatical errors should be corrected at revision, so please note any specific errors here.

Reviewer #2: Yes

6. Review Comments to the Author

Please use the space provided to explain your answers to the questions above and, if applicable, provide comments about issues authors must address before this protocol can be accepted for publication. You may also include additional comments for the author, including concerns about research or publication ethics.

You may also provide optional suggestions and comments to authors that they might find helpful in planning their study.


(Please upload your review as an attachment if it exceeds 20,000 characters)

Reviewer #2: I will focus on methods and reporting, as the statistical reviewer. Methods and approach to the sample are appropriate. I only have a couple of minor points to raise.

1) eligibility criteria. There are numerous ongoing interventions and the participants need to be at least asked about such participation so that can be included in the analyses (rather than as an additional exclusion criterion).
2) In the UK, under the Quality and outcomes framework, HbA1c targets are incentivised at the practice level. Does that run a risk of contaminating the trial in any way? Treatment information will be collected the authors say, but needs to be collected very well and possibly not be self-reported. Detailed information is needed in this space.

Author response:

6 – 1) Thank you for your comments.

We have added a sentence under the eligibility sub-heading to clarify that eligible individuals will be invited to take part and that, where declined, reasons for decline will be recorded (lines 148, 180, 183).

6 – 2) HbA1c target incentivisation was considered as a potential confounder in this trial, however with recruitment occurring from a secondary care diabetes clinic, primary care target incentivisation during the study period is not anticipated to influence study results. Certainly, if recruitment was also being undertaken from primary care, we would need to account for this. We have updated the manuscript for clarification (line 160).

The authors agree that the collection of treatment information must be thorough and accurate. These data will be obtained from medical records rather than relying on self-reported data. We have updated the manuscript to clarify this (line 242).

7. PLOS authors have the option to publish the peer review history of their article ([what does this mean?](https://eur01.safelinks.protection.outlook.com/?url=https%3A%2F%2Fjournals.plos.org%2Fplosone%2Fs%2Feditorial-and-peer-review-process%23loc-peer-review-history&data=05%7C01%7C23723122%40edgehill.ac.uk%7Cdbeec7f19a7d4497e44608da6e659082%7C093586914d8e491caa760a5cbd5ba734%7C0%7C0%7C637943679743277065%7CUnknown%7CTWFpbGZsb3d8eyJWIjoiMC4wLjAwMDAiLCJQIjoiV2luMzIiLCJBTiI6Ik1haWwiLCJXVCI6Mn0%3D%7C3000%7C%7C%7C&sdata=42KZ3fL8lJKvJh4OHQUwsmjavCnqWJLpl%2BlLxo42IFs%3D&reserved=0)). If published, this will include your full peer review and any attached files.


If you choose “no”, your identity will remain anonymous but your review may still be made public.


**Do you want your identity to be public for this peer review?** For information about this choice, including consent withdrawal, please see our [Privacy Policy](https://eur01.safelinks.protection.outlook.com/?url=https%3A%2F%2Fwww.plos.org%2Fprivacy-policy&data=05%7C01%7C23723122%40edgehill.ac.uk%7Cdbeec7f19a7d4497e44608da6e659082%7C093586914d8e491caa760a5cbd5ba734%7C0%7C0%7C637943679743277065%7CUnknown%7CTWFpbGZsb3d8eyJWIjoiMC4wLjAwMDAiLCJQIjoiV2luMzIiLCJBTiI6Ik1haWwiLCJXVCI6Mn0%3D%7C3000%7C%7C%7C&sdata=ok8T%2FON9FNBk541aTBgf3jA9OX2zQ3OY6VH%2F%2FIGKbSc%3D&reserved=0).

Reviewer #2: No

------END OF RESPONSE------
